# Graphene Visualizes the Ion Distribution on Air-Cleaved Mica

**DOI:** 10.1038/srep43451

**Published:** 2017-03-06

**Authors:** Pantelis Bampoulis, Kai Sotthewes, Martin H. Siekman, Harold J. W. Zandvliet, Bene Poelsema

**Affiliations:** 1 Physics of Interfaces and Nanomaterials, MESA+ Institute for Nanotechnology, University of Twente, P.O. Box 217, 7500AE Enschede, The Netherlands; 2 Physics of Fluids and J.M. Burgers Centre for Fluid Mechanics, MESA+ Institute for Nanotechnology, University of Twente, P.O. Box 217, 7500AE Enschede, The Netherlands

## Abstract

The distribution of potassium (*K*^+^) ions on air-cleaved mica is important in many interfacial phenomena such as crystal growth, self-assembly and charge transfer on mica. However, due to experimental limitations to nondestructively probe single ions and ionic domains, their exact lateral organization is yet unknown. We show, by the use of graphene as an ultra-thin protective coating and scanning probe microscopies, that single potassium ions form ordered structures that are covered by an ice layer. The *K*^+^ ions prefer to minimize the number of nearest neighbour *K*^+^ ions by forming row-like structures as well as small domains. This trend is a result of repulsive ionic forces between adjacent ions, weakened due to screening by the surrounding water molecules. Using high resolution conductive atomic force microscopy maps, the local conductance of the graphene is measured, revealing a direct correlation between the *K*^+^ distribution and the structure of the ice layer. Our results shed light on the local distribution of ions on the air-cleaved mica, solving a long-standing enigma. They also provide a detailed understanding of charge transfer from the ionic domains towards graphene.

Muscovite mica has been the substrate of choice for a large number of scientific studies and technological applications owing to its atomically flat surface, high surface energy, low thermal and electrical conductivity and its perfect cleavage plane along the (0001) direction^1^,^2^. Muscovite mica has a layered aluminosilicate structure with the formula *KAl*_2_*(Al,Si*_3_*)O*_10_*(OH)*_2_. The structure of mica consists of an octahedrally coordinated *Al*^3+^ sheet which is sandwiched between two tetrahedrally coordinated sheets of *Al*^3+^ and *Si*^4+^, see Fig. 1a,b. The random substitution of *Si*^4+^ by *Al*^3+^ in the tetrahedral sheet results in a negative charge. Interlayer potassium cations hold together these 2:1 layers and balance the negative charge of the mica crystal. After cleavage of the mica, half of the potassium ions are expected to remain on each of the two created surfaces in order to preserve charge balance. The atomic lattice periodicity of mica has been obtained by Atomic Force microscopy (AFM) and Friction Force Microscopy (FFM)^2^,^3^,^4^,^5^. However, little is known about the position and lateral organization of the potassium ions of the air-cleaved mica. Reliable and atomically resolved scanning probe images at ambient conditions are hard to obtain because of the danger of causing irreversible changes in the lateral organization of the *K*^+^ ions. In many cases the large forces exerted on the surface result in significant damage and loss of lateral resolution.

Although scanning probe techniques have been extensively used to study the surface of mica immersed in aqueous ionic solutions^6^,^7^,^8^, such techniques are often not applicable to ambient conditions studies. Remarkably, atomic resolution images are readily obtained owing to the reduction of the exerted AFM tip forces^9^,^10^. In a recent study, Ricci *et al*.^11^ used interfacial dissipation microscopy to image different metal ions adsorbed at the surface of mica when immersed in water. Surprisingly, they observed that the ions do not adsorb randomly, but prefer to form ordered arrangements. The formation and stabilization of these structures is apparently achieved by minimizing the surface energy of water at the mica-ion interface.

Understanding the complexity of the lateral organization of ions on air-cleaved mica surface is key in crystal growth and self-assembly of molecules on mica and justifiably has received intense scientific interest^1^. Most theoretical studies assume a periodic structure of potassium cations. Nonetheless this is a crude assumption since a uniform distribution of *K*^+^ ions is unlikely due to the randomness of the cleaving process. Other studies suggest the formation of *K*^+^ islands, leaving thus ion-free mica regions^12^. However, due to the repulsive ion-ion interaction and the tendency to achieve electro-neutrality on the surface, we consider this scenario contrary to what is expected.

Here we use graphene as an ultra thin coating for enabling the visualization of single ions and ionic clusters on the mica surface. Graphene has been shown to be able to accurately stabilize and conform to water layers^13^,^14^,^15^,^16^,^17^,^18^,^19^,^20^ and other small molecules^21^,^22^,^23^,^24^ when deposited on a substrate. It has been proven an essential tool to probe small and otherwise dynamic molecules. We show by means of scanning tunneling microscopy (STM) and Lateral Force Microscopy (LFM) that graphene can enable non-destructive visualization, with single-ion resolution, of the lateral organization of ions on the surface of air-cleaved mica. We find that the potassium ions spontaneously form ordered arrangements, i.e. rows and small hexagonal domains, on the surface. The presence of potassium ions at the mica surface is favored by the system’s preference for charge neutrality and probably enhanced by the graphene cover sheet. The lateral distribution of the ions is then governed by repulsive Coulomb interactions with a range reduced by the surrounding water molecules. Conductive (or often referred as current sensing) AFM (C-AFM) maps with adequate lateral resolution show direct correlation between the *K*^+^ distribution and the conductance of graphene. Immediately above the *K*^+^ ions, graphene is significantly less conductive than over ion-free locations. This result indicates charge transfer from the underlying surface to graphene and is in good agreement with previous studies^15^,^25^,^26^,^27^. Our results shed light on the local distribution of ions on air-cleaved mica and pave the way for controlling the local electronic landscape of graphene.

## Results and Discussion

**Figure 1:**
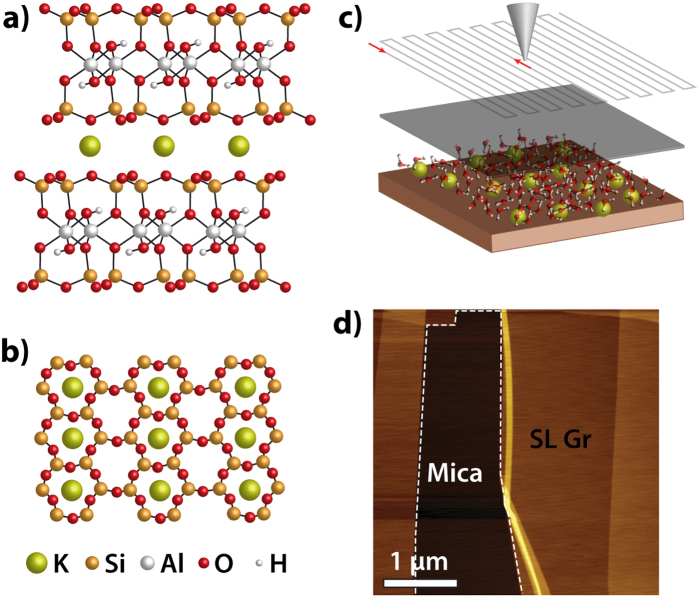
The crystal structure of mica and graphene deposition. (**a**) Top and (**b**) side view of the crystal structure of muscovite mica. Note here that the exact distribution of the *K*^+^ ions is not yet known. (**c**) An overview of the experimental procedure. Graphene covers the freshly cleaved mica surface. Water molecules co-exist with potassium ions. The graphene surface can be scanned by the use of an AFM which allows to non-destructively obtain information about the confined nanostructures. (**d**) An AFM topograph of a graphene flake deposited on the mica surface. The region of single layer (SL) graphene is indicated. The white dashed line indicates the borders of the graphene flake.

**Figure 2:**
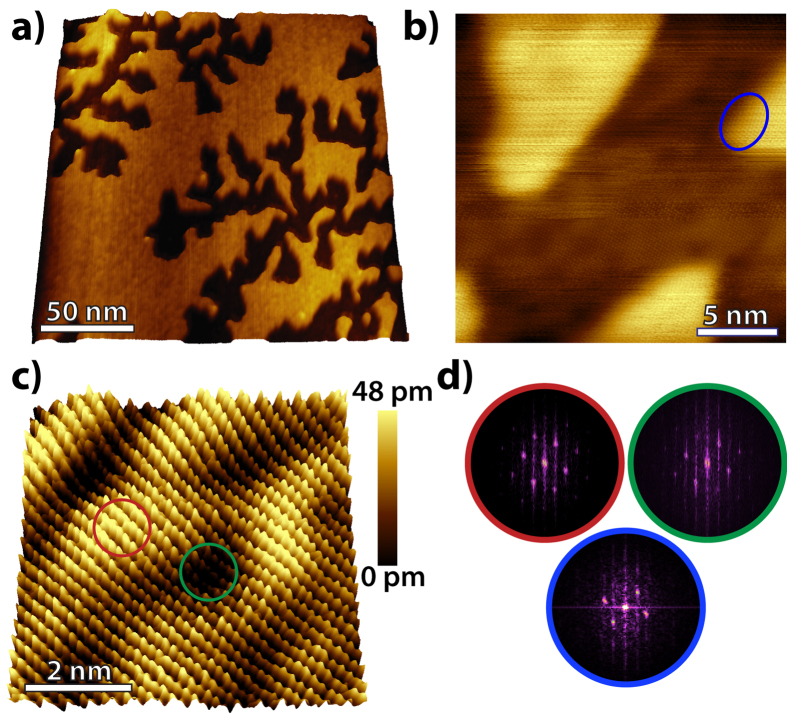
Scanning tunneling microscopy of the Graphene-Ice-Mica interface. (**a**) UHV STM topography (190 × 190 *nm*^2^) of a few layers graphene deposited on mica recorded at 0.2 V and 100 pA. Ice crystals (darker regions) are observed intercalated between graphene and mica surrounded by two water layers (brighter region). (**b**) A high resolution image (17 × 17 *nm*^2^) at the edges between an ice crystal and two water layers. A ripple-like structure is observed. (**c**) An atomic resolution image (6 × 6 *nm*^2^) clearly showing the ripple-like structure of graphene. (**d**) FFT of the hills (red circle in panel (c)) and valleys (green circle in panel (c)) of the rippled graphene surface and the higher edges of the fractal (blue circle in panel (b)).

Freshly cleaved mica is reactive and immediately a thin water film is adsorbed and covers its entire surface^28^. When graphene is placed on mica, this water film assumes a defined structure as a result of the confinement^14^,^15^. It consists of two water layers with thickness close to that of the interlayer distance of bilayer hexagonal ice, i.e. *I*_*h*_-ice. A cartoon of the experimental system is shown in Fig. 1c. An AFM topographic image of single layer graphene on mica is shown in Fig. 1d. After preparation the samples were immediately inserted into an ultra high vacuum (UHV) chamber, where the STM measurements were performed. Ice crystals such as the one shown in Fig. 2a (dark areas) are found all over the surface. They are located underneath the graphene cover and are one layer thinner than their brighter surroundings (double water layer). The growth of the ice crystals is induced by the heat extracted from the system by evaporation of intercalated water molecules, from the graphene-mica interface, into the ambient under low humidity^15^. Due to the small scan size of STM (5 × 5 *μm*^2^), we are unable to determine the exact number of graphene layers. Nevertheless from the size of the observed fractals we do not expect the thickness to be larger than 4 graphene layers^29^.

Small scale STM images, Fig. 2b and c, reveal a ripple-like roughness of the graphene surface that is best visible at the interior of the ice crystals. For clarity, the RMS roughness at the interior and the surroundings of the ice crystal is 50 *pm* and 35 *pm*, respectively. The difference of the RMS roughness between the two regions is a result of the extra water layer at the environment of the ice crystals, which can smoothen the interface by filling atomic scale defects. The obtained RMS values are larger than those reported in earlier AFM studies^14^,^30^. We attribute this discrepancy to the higher (-atomic) resolution of our STM images, which compared to the large scale AFM images of those earlier studies give a much more reliable roughness measurement. The rippling periodicity and amplitude observed here amounts to 1–2 nm and 50 pm, respectively. The height of the protrusions fits well with heights reported for ions adsorbed on mica when immersed in aqueous ionic solutions^6^. The apparent lattice constant, as obtained by Fast Fourier Transforms (FFT), differs by 2% between the hills and valleys of the rippled graphene surface (see Fig. 2d). The difference is attributed to local stretching of the graphene near the hill tops and local compression of the graphene sheet near the valleys when it is conformed to the structures underneath. In contrast, the difference reaches about 8% at the edges of the ice crystals, indicating that the ripples are real topographic features. We consider the formation of ripples as evidence for the existence of potassium ions nonuniformly distributed on the mica surface. Unfortunately, convolution induced by the few layers graphene cover does not allow identification of single ions at the interface.

**Figure 3:**
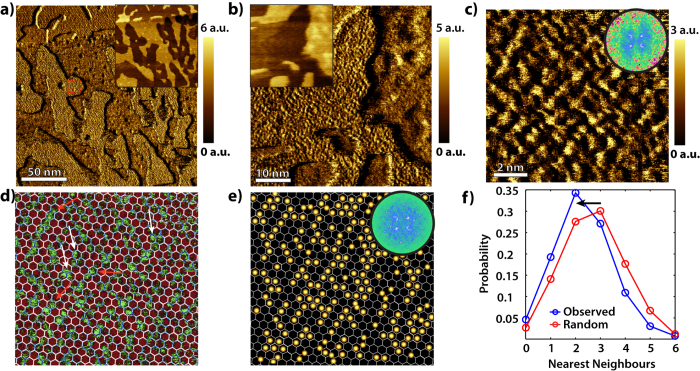
Lateral force Microscopy of the Graphene-Ice-Mica interface. (**a**) Lateral force image (200 × 200 *nm*^2^) of an ice crystal intercalated between graphene and mica. The graphene situated just above the ice crystal displays a ripple-like structure. Inset: the corresponding topographic image. (**b**) A small scale lateral force image (45 × 45 *nm*^2^) of the structure observed in the fractal. Inset: the corresponding topographic image. (**c**) A zoomed in lateral force image (15 × 15 *nm*^2^) of graphene directly above an ice crystal (at the location indicated with the red box in (**a**)). Single ions are observed forming row-like structures and small domains. Inset: The corresponding FFT. (**d**) Filtered image of (**c**) using an appropriate threshold and overlaid with a hexagonal grid. Row-like and small domain structures are indicated with red and white arrows respectively. (**e**) The locations occupied by single ions obtained from (**d**) and overlaid with a hexagonal grid. Inset: The corresponding FFT. (**f**) Histograms of the nearest neighbours distribution of single ions on mica (N = 1220, mean = 2.32) and a random distribution (N = 1681, mean = 2.73) on an identical lattice obtained via equation 1.

In order to overcome this problem, we have performed LFM on a single-layer (SL) graphene-mica interface under low humidity conditions. Note that LFM can resolve atomic lattice periodicities of solid surfaces^4^,^5^. We have used forces in the order of 1 *nN* in order to avoid any irreversible damage of the surface. Lateral force images, Fig. 3a and b, recorded simultaneously with topographic (deflection) images (see insets of Fig. 3a and b) reveal a rough surface at the location of the ice crystals. The mean lateral force value of graphene above the ice crystals is substantially higher than that of the surroundings. The surface above the ice crystals has a ripple-like structure similar to that observed by STM. The same ripple-like structure is also present in the surroundings of the ice crystals, i.e. 2 layers of water, however less pronounced. This is due to convolution effects of the 2 water layers. Small scale friction images recorded directly above an ice crystal (red dashed box in Fig. 3a), Fig. 3c, were subsequently obtained and reveal bright protrusions. Interestingly, the observed protrusions appear to follow the mica lattice and have a hexagonal arrangement as is revealed by the recorded FFT. The separation distance between adjacent protrusions was found to be equal to the lattice constant of mica, i.e. 0.52 *nm*. The protrusions unambiguously indicate the presence of *K*^+^ ions at the graphene-mica interface. Surprisingly, the ions appear to preferentially form rows and small clusters. A reference mica lattice can be reconstructed by FFT and overlayed with the locations of the *K*^+^ ions, Fig. 3d and e. The variation in the protrusions’ brightness is indicative of variations in the adsorption site on the underlying surface, i.e. mica inner hexagonal cell, atop *Si*^4+^ or *Al*^3+^. In agreement with our observations, Loh *et al*.^6^ demonstrated that indeed the height contrast of the ions depends on their exact binding sites. Unfortunately, our measurements cannot determine the exact lattice position of the ions on the mica surface. Furthermore, successive AFM images reveal that the *K*^+^ ions are stable (see supplementary information), demonstrating the conserving role of the graphene cover against the applied tip force.

From the distribution of *K*^+^ ions a histogram of nearest neighbours is extracted and reveals a preference for having 2 and 3 neighbours (see Fig. 3f). This corresponds very well to the number of neighbours expected for row-like and very small domains with a hexagonal base pattern. From several images, like the one in Fig. 3c, at different locations and different samples, we found that the surface coverage of *K*^+^ ions is approximately 45%, i.e. very close to the expected 50% coverage of air-cleaved muscovite mica. The observed distribution deviates from a random distribution in which the ions adopt a two-dimensional gas-like arrangement, as shown in Fig. 3f. The random distribution probability is calculated by considering a hexagonal lattice and no interactions between the particles. The probability to find a particle with *nn* number of nearest neighbours is given by:


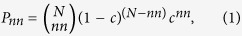


where *N* is the number of particles and *c* is the coverage. The only variable parameter is the coverage, which is extracted from the lateral force images, i.e. approximately 45%. The histograms reveal that the adsorbed ions on the mica tend to minimize the number of nearest neighbour *K*^+^ ions, compared to ions that are randomly distributed on an identical lattice. This finding is consistent with repulsive interactions between the positive ions. Interestingly, the shift to lesser nearest neightbours is contrary to the findings of Ricci *et al*.^11^ for adsorbed ions on mica in aqueous ionic solutions.

The results of Ricci *et al*.^11^, mentioned further above, are explained due to attractive ion-ion interactions. They found that the organization of these geometrically closed packed ionic structures is controlled by the organization of water molecules at the interface. The water molecules adjacent to the adsorbed ions obtain better coordination in the hydration structure of neighbouring ions as opposed to single ions. As a result the hydration free energy is minimized and dominates the repulsive electrostatic forces. In contrast to Ricci *et al*.^11^ our system is not immersed in water. Although, water layers do exist in the graphene-mica interface, they apparently play a less dominant role in the positioning of the potassium ions. The shift to lesser nearest neighbours in the ion distribution in the current case can only be explained by the repulsive ion-ion interactions. On the freshly air-cleaved mica (immediately after cleavage), the *K*^+^ ions repel each other and therefore try to minimize the amount of nearest neightbours. Because of the high coverage it is not possible for the ions to achieve zero nearest neighbours and therefore row-like and small hexagonal domains are formed. The system’s preference for charge neutrality further favors the presence of *K*^+^ ions. The lateral distribution of the ions is governed by repulsive Coulomb interactions with a range that is modified by the surrounding water molecules. With the help of Monte Carlo simulations (see supplementary information) this scenario is tested. We have considered the adsorption of ions with well-defined interactions ranging from highly repulsive to no interaction between neighbouring ions. Our experimental results fit well with the simulations when moderate repulsion between neighbouring ions is considered. The relative weakness of the repulsive interactions is a result of the presence of water which reduces the range of the Coulomb repulsion.

**Figure 4:**
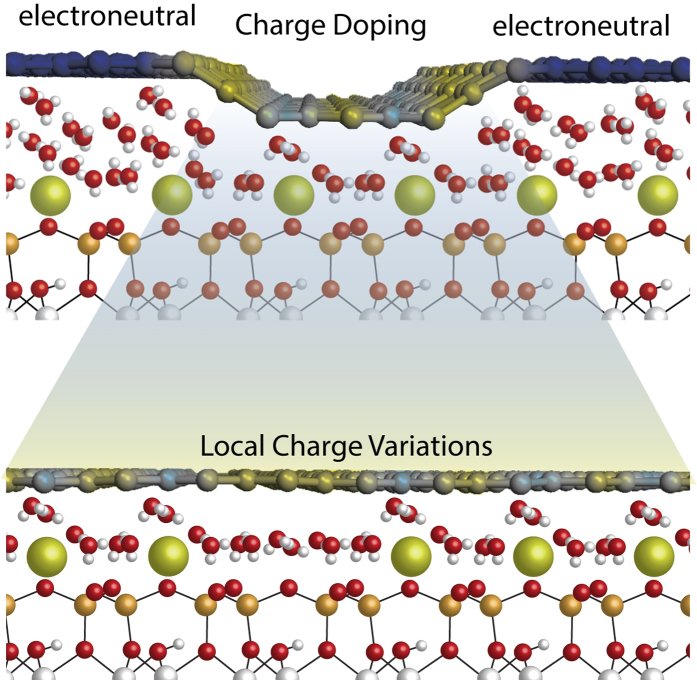
A schematic representation of the graphene-mica interface. Water molecules form order structures at the interface. At the locations of the ice crystals the water molecules, form a crystalline structure of ice that is polarized^30^. This ice layer is responsible for charge transfer onto the graphene cover. Local charge variations are induced by missing potassium ions.

Previous studies have demonstrated^15^,^25^,^26^,^27^ that graphene in contact with a supporting substrate can be substantially doped. When in contact with the mica surface graphene conserves its unique properties^27^, even when mica has been shown to induce doping of graphene^15^,^26^. In a previous study we have shown that graphene above the double water layer is only slightly n-type doped. This indicates that the double water layer does not lead to a sizable charge transfer. Surprisingly, graphene above the ice crystals/lower level fractals is p-type doped. The potassium ions electronically neutralize the mica and therefore should not induce any significant doping on the graphene cover^27^. The doping is rather caused by an ice layer covering the mica-*K*^+^ surface, similar to that of Fig. 4 (the ice structure has been taken from ref. 31). In this ice structure, the OH bonds face either the mica surface or in plane water molecules^31^ and therefore graphene effectively lies on a negatively charged surface.

**Figure 5:**
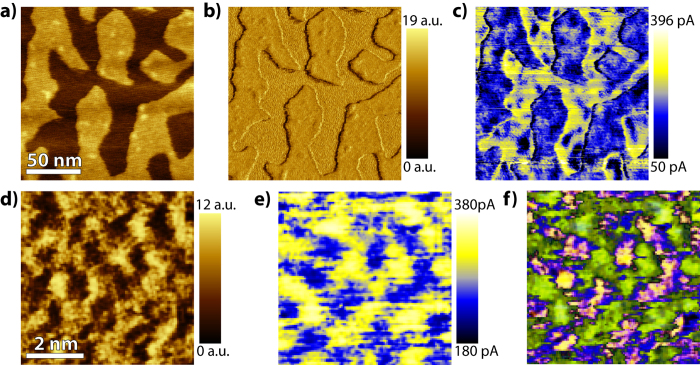
The graphene conductance and its relation to the ion distribution. (**a**) Topography, (**b**) lateral force and (**c**) conductance image (150 × 150 *nm*^2^) of graphene above an ice crystal on mica. The conductance image (**c**) clearly shows that the conductance of graphene above the ice crystal is higher than above the two water layers. (**d**) A small scale (6 × 6 *nm*^2^) lateral force image of graphene above an ice crystal. Potassium domains (bright) are observed. (**e**) The corresponding conductance map of (**d**) showing the regions where potassium is missing are more conductive. (**f**) An overlay of (**d**) and (**e**), showing strong correlation between the locations of potassium vacancies and the higher conductance regions (green regions). The voltage bias was 1 *V* for all recorded images presented here.

In order to confirm the existence of an ice-like layer covering a nonuniform distribution of *K*^+^ ions in the fractal regions, conductive AFM (C-AFM) is used. In C-AFM a conductive tip is maintained in mechanical contact with the surface while it measures the current generated by the applied bias voltage. Figure 5a is an AFM topography image of graphene covering an ice crystal. The corresponding lateral force image is shown in Fig. 5b. The detailed structure induced by the *K*^+^ ions is found inside the fractal. The simultaneously recorded conductance map is given in 5c. As expected, the regions above the double layer of water are substantially less conductive than the graphene regions just above the ice crystals. Since the conductance depends on the local electron density, i.e. the density of states around the Fermi level, the doped graphene shows a higher conduction because of a higher density of states^15^,^27^. The heterogeneities in the conduction map are mainly a result of small droplets found occasionally at the interface. The higher conductance is a result of the doping of the graphene just above the ice crystal. In the absence of ice crystals, the conductance of the graphene remains unaltered^32^.

Small scale high resolution friction (Fig. 5d) and the first ever conduction maps with resolved ions (Fig. 5e) reveal that graphene in the troughs of adjacent *K*^+^ rows (vacancies) exhibit higher electrical conductance as compared to graphene directly above the ions. The correlation becomes better visible by overlaying the two images, as shown in Fig. 5f. Strong correlation is observed between the darker regions of the lateral force image (potassium vacancies) (Fig. 5d)) and the high conductance regions (yellow) in the conductance map (Fig. 5e). The differences in the conductance of graphene inside the fractal is a result of local variations in the charge transfer from the underlying substrate. As mentioned previously, the ice layer is polarized, resulting in a negatively charged surface^31^ (see Fig. 4). In addition, the mica surface without the *K*^+^ ions is negatively charged, whereas the mica surface with *K*^+^ ions is neutral. At the positions where the *K*^+^ ions are located the negatively charged surface is induced by the polarized ice layer. However, at locations where the *K*^+^ ions are missing, the negatively charged surface is induced by the ice layer as well as the mica surface. Therefore the local electron density is higher and a higher conductance is measured at the locations were *K*^+^ ions are missing. The small overlap (blue region in Fig. 5) is a result of small charge spread and it is only limited at the edges of the ionic domains. These results clearly demonstrate the existence of a nonuniform ion distribution that is covered by an ice layer. The existence of a nonuniform distribution of *K*^+^ ions is of high significance in a plethora of studies where mica is used as a substrate, e.g. biology, crystal growth and surface engineering. Our findings may bear important implications for the interpretation of earlier experimental studies which disregarded the possibility of non uniformly distributed *K*^+^ ions on mica in contact with a polarized ice layer^13^,^26^.

## Conclusions

Using scanning probe microscopies in combination with graphene as an ultra thin cover, we have managed to obtain information on the local organization of ionic domains on air-cleaved mica surfaces. Lateral force images show that the *K*^+^ ions prefer to reduce the number of nearest neighbours (coordination number) due to repulsive ion-ion interactions by forming specific arrangements. The repulsive ion-ion forces are screened by the presence of ordered water layers. The ordered water/ice-*K*^+^ interface is studied by measuring the electronic properties of the graphene cover using conductive AFM. At locations where the water molecules arrange in a fractal-like crystalline structure, graphene has a higher electrical conductance due to the higher local electron density induced by charge transfer from the ice layer. High resolution conductance images reveal single ions and and show distinct variations of the conductance exactly above the ice layer. These variations show a direct correlation with the nonuniform distribution of *K*^+^ ions. The current results are of importance in crystal growth and self-assembly on mica surfaces and give new valuable information on charge transfer phenomena in the graphene cover.

## Methods

Graphene was obtained from highly oriented pyrolytic graphite (HOPG ZYA grade, MikroMasch) through the microexfoliation process and immediately deposited on a freshly cleaved mica surface (SPI, V1). Optical Microscopy (DM2500 MH materials microscope, Leica, Germany) and tapping mode Atomic Force Microscopy, AFM (Agilent 5100 atomic force microscope, Agilent) were used to determine the thickness of graphene^33^. Lateral Force Microscopy (LFM) and Conductive AFM (C-AFM) imaging of the Gr-mica system was performed at room temperature and in contact mode using AD-E-0.5-SS tips (diamond tips, Adama innovations) with a nominal spring-constant of 0.5 *N/m* and resonance frequency of 30 *kHz*. The AFM scanner was placed inside an environmental glove box, in which the relative humidity (RH) could be controlled. The RH was tuned by an adjustable *N_2_* flow and was measured using a humidity sensor (SENSIRION EK-H4 SHTXX, Humidity Sensors, Eval Kit, SENSIRION, Switzerland), with an accuracy of 1.8% between 10–90% RH. Scanning tunneling microscopy (STM) was performed in an RHK Technology UHV3000 variable temperature STM operating at a base pressure of 1 × 10^−10^ *mbar*, using chemically etched W tips. All the STM images are aquired at room temperature. For the STM and C-AFM measurements the graphene flake was mechanically connected with a bigger graphite flake acting as an electrode.

## Additional Information

**How to cite this article:** Bampoulis, P. *et al*. Graphene Visualizes the Ion Distribution on Air-Cleaved Mica. *Sci. Rep.* **7**, 43451; doi: 10.1038/srep43451 (2017).

**Publisher's note:** Springer Nature remains neutral with regard to jurisdictional claims in published maps and institutional affiliations.

## Supplementary Material

Supplementary Information
