# Supplementary material for: Graphene Visualizes the Ion Distribution on Air-Cleaved Mica
Source: Sci Rep. 2017 Mar 6;7:43451. doi: 10.1038/srep43451 (PMC5337958; doi:10.1038/srep43451)
Supplement: Supplementary Information [file srep43451-s1.pdf]

Graphene Visualizes the Ion Distribution on Air-Cleaved Mica

Pantelis Bampoulis, Kai Sotthewes, Martin H. Siekman, Harold J. W. Zandvliet & Bene Poelsema

1 Non destructive imaging

Graphene not only enables the visualization of single ions and ionic domains but it can also protect them from the probing tip. We have performed consecutiveLFM imaging of graphene over an ice crystal using a force of about 1 nN. Figure S1 shows two consecutive images recorded at the same location. Little to no changes are observed between the two images, i.e. the ions remain at their initial location. The exerted tip forces are screened by the graphene cover.

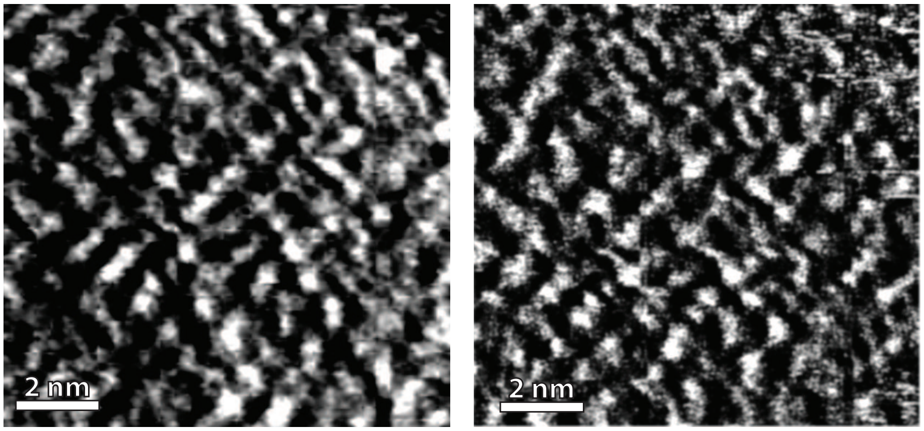

Figure S1: (a) and (b) are consecutive friction images recorded at the same location. The load exerted by the AFM tip was calculated to be around 1 nN. These images reveal that the graphene enables non-destructive visualization of single ions and ionic domains.

2 Random Distribution

The analytical expression describing a random distribution on a hexagonal grid when no interactions are taken into account, is obtained by assuming a single hexagonal lattice where the middle position is occupied. For zero number of nearest neighbours non of the surrounding positions can be occupied. The chance per position, that the position is unoccupied, is (1-c), where c is the coverage. There are in total six positions, thus the total probability is $P_0 =$

$(1-c)^6$. For the case of one nearest neighbour, five of the positions are available while one position is occupied. Therefore the probability is given by $(1-c)^5c$. However, there are 5! ways to choose a subset of n elements, disregarding their order, from a set of N elements. N is the amount of positions, i.e. 6 and nn is the number of nearest neighbours. This leads to the following expression:

$$P_1 = \binom{6}{1}(1-c)^5c^1, \tag{1}$$

for two nearest neighbours the probability is:

$$P_2 = \binom{6}{2}(1-c)^4c^2, \tag{2}$$

When this is generalized for all the numbers of nearest neighbours, we arrive at the final expression given by equation 3:

$$P_{nn} = \binom{N}{nn}(1-c)^{N-nn}c^{nn}, \tag{3}$$

where N is the amount of particles, c is the coverage and nn is the number of nearest neighbours. With the coverage, extracted from the lateral force images, the random distribution can be calculated.

3 Monte Carlo Simulations

We have performed Monte Carlo (MC) simulations in order to obtain information on the nature of the ion-ion interactions on the air cleaved mica. The simulations were performed by assuming a hexagonal grid. The lattice sites of the grid are available positions for particles (ions) to occupy. Initially a particle occupies a random site on the hexagonal grid with a probability P_o. The next generated particle will have a probability of occupying a lattice site P_o away from the first generated particle, i.e. locations where the coordination number/nearest neighbours is 0. At locations where nn is 1, i.e. the sites next to the first generated particle, the probability for occupying these sites drops to $P = P_o^{1+nn} = P_o^2$. Similarly for N generated particles, the N+1 particle will have a probability to occupy a site of the hexagonal grid equal to $P = P_o^{1+nn}$, where nn is the nearest occupied particles/ neighbours of this particular grid site. Here P_o represents the strength of the interactions. It can take values between 0, the particles infinitely repel each other, and 1, representing no interactions between the particles. For the case of $P_o = 1$ every grid site can be equally occupied by the generated particles, leading to a random distribution. The obtained distribution is perfectly described by the aforementioned eq. 3. When P is between 0 and 1, the probability depends on the amount of nearest neighbours of a particular site.

The particles are generated one by one up until the coverage reaches 45%, i.e. equal to the coverage obtained experimentally. We note here that in the

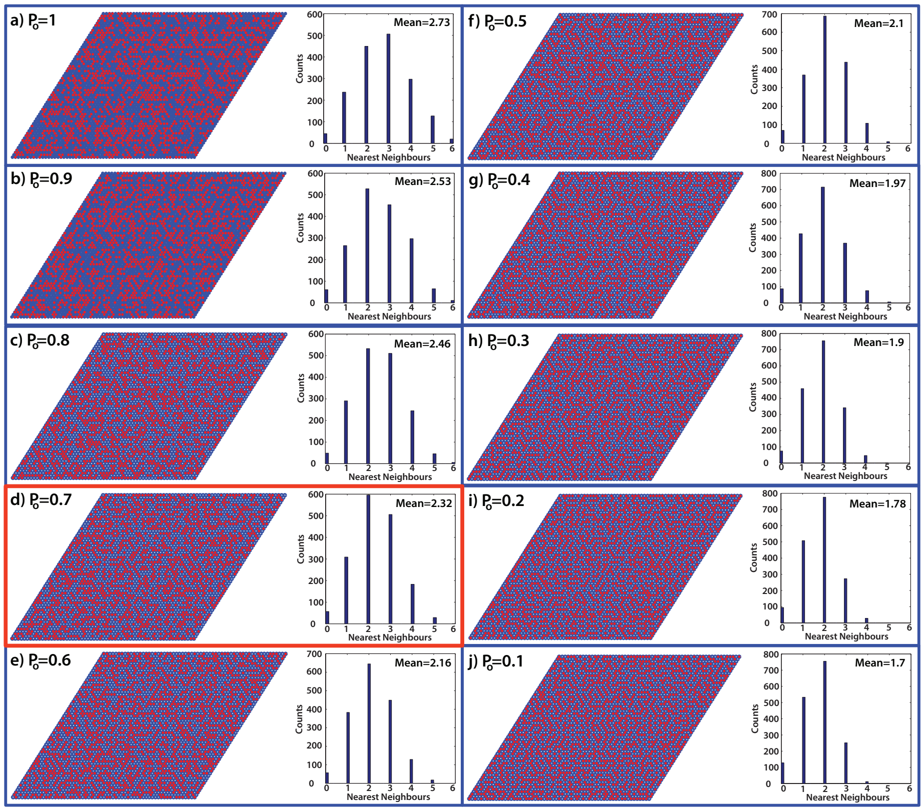

Figure S2: The organization and histogram of the probability distribution of 1681 generated particles on a hexagonal grid for (a) $P_o = 1$, (b) $P_o = 0.9$, (c) $P_o = 0.8$, (d) $P_o = 0.7$ (the red box indicates that the obtained distribution matches with the experimental observations), (e) $P_o = 0.6$, (f) $P_o = 0.5$, (g) $P_o = 0.4$, (h) $P_o = 0.3$, (i) $P_o = 0.2$, (j) $P_o = 0.1$

experimental systems the ions are not generated one by one but they pre-exist on the mica surface. Another difference is related to the mobility of the ions which is finite. After cleavage one should expect that the ions can up to some extent diffuse across the mica and acquire a more favorable configuration. For simplicity in the simulations the ions are static and cannot reorder themselves. Therefore our MC simulations should be considered as a qualitative or at most a semi-quantitative attempt to identify the nature of the lateral interactions between the ions.

The results for discrete values of P_o range from 1 to 0.1 are presented in figure S2. Images of each of the generated distributions and their corresponding probability distribution histograms involving 1681 particles are shown in figure S2. The simulations with $P_o = 0.7$ excellently reproduce the experimental images and distribution probability, shown in the main text, with a mean value of nearest neighbours around 2.3. This illustrates that the measured arrangement of ions on the air cleaved mica is a result of moderate repulsion between adjacent ions.

Statistical analysis of the nearest neighbours of each potassium ion reveals a distribution that looks like figure 3. There is a clear preference for 2 nearest neighbours. We compare this distribution with a random distribution. The random distribution probability is generated using the mica lattice structure and a coverage of 45%, i.e. the same as the experimental distributions. The random distribution probability is a typical Gaussian distribution as expected for a coverage of 45% of the available sites. A clear difference is observed between the two distributions. A shift of the maximum occurs for the nearest neighbours of the potassium ions towards the left hand side. This is interpreted as indicative of weak repulsion between adjacent ions with its weakness caused by the screening ability of the water molecules.

4 Graphene Convolution

The uniformity of graphene can influence the experimental results. For that reason, we have used high quality graphene that is obtained by mechanical exfoliation of Highly Oriented Pyrolytic graphite ZYA (highest possible quality), see the experimental methods for further details on the preparation procedure. This way we have limited the presence of defects such as folds and wrinkles. We have also limited our experiments to consider only defect free locations, i.e. locations away from any possible wrinkles or folds. Secondly, graphene flakes of different thicknesses have been easily obtained throughout our samples. We have chosen to only consider images were graphene is a single layer in our analysis. However, regions of thicker graphene can be also readily obtained. Note here, that a thicker graphene cover will lead to larger convolution effects. Since the conformity of graphene is decreasing as a function of each thickness, thicker graphene will not be able to accurately conform the underlying interface. Therefore, information regarding the potassium distribution will be more difficult to obtain if not impossible. For example, for bilayer graphene in order to be able to

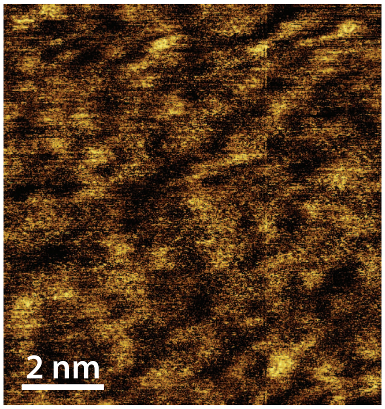

Figure S3: An LFM image of a bilayer graphene on the ice-K^+/mica interface. The load exerted by the AFM tip was calculated to be around 25 nN. Single ions are difficult to be identified as compared to single layer graphene due to the extra convolution induced by the thicker graphene cover.

visualize ions and ion clusters, we need to use higher forces during scanning. It is also more difficult to distinguish between single ions compared to single layer graphene due to this extra convolution. This is demonstrated in figure S3, a bilayer graphene is shown above the ice-K^+ interface. In contrast to the single layer graphene, the obtained image appears to be more convoluted. Thicker graphenes (>5 layers) display no apparent contrast in LFM images, even after scanning with very high forces (50 nN), which shows that the graphene cover doesn't anymore accurately (nanoscopic precision) conform the underlying surface.
